# Supplementary material for: Trace metals and their human health risks in sesame seeds from the main cultivation areas of Ethiopia
Source: PLoS One. 2025 Mar 7;20(3):e0319566. doi: 10.1371/journal.pone.0319566 (PMC11888145; doi:10.1371/journal.pone.0319566)
Supplement: Attachment — Submitted filename: Author Response.docx [file pone.0319566.s001.docx]

PONE-D-24-46643

Trace metals and their human health risks in sesame seeds from the main cultivation areas of Ethiopia

Dear Muhammad Sibt-e-Abbas
Academic Editor
PLOS ONE,

**Author Response**: We thank the editor and the three reviewers for reviewing our manuscript favorably.

**Journal Requirements:**

1. When submitting your revision, we need you to address these additional requirements.

Please ensure that your manuscript meets PLOS ONE's style requirements, including those for file naming. The PLOS ONE style templates can be found at 
https://journals.plos.org/plosone/s/file?id=wjVg/PLOSOne_formatting_sample_main_body.pdf and 
<https://journals.plos.org/plosone/s/file?id=ba62/PLOSOne_formatting_sample_title_authors_affiliations.pdf>

**Author Response**: We thank the editor for handling our manuscript favorably. We have followed PLOS ONE’s style requirements.

2. In your Methods section, please provide additional information regarding the permits you obtained for the work. Please ensure you have included the full name of the authority that approved the field site access and, if no permits were required, a brief statement explaining why.

**Author Response**: Samples were collected from private farmlands, after permission was granted by the farmers to access their fields. This information is now provided in the method section.

3. We note that your Data Availability Statement is currently as follows: All relevant data are within the manuscript and its Supporting Information files.

Please confirm at this time whether or not your submission contains all raw data required to replicate the results of your study. Authors must share the “minimal data set” for their submission. PLOS defines the minimal data set to consist of the data required to replicate all study findings reported in the article, as well as related metadata and methods (<https://journals.plos.org/plosone/s/data-availability#loc-minimal-data-set-definition>).

**Author Response**: We confirm that the submission contains all raw data required to replicate the results of the study.

For example, authors should submit the following data:

- The values behind the means, standard deviations and other measures reported;
- The values used to build graphs;
- The points extracted from images for analysis.

Authors do not need to submit their entire data set if only a portion of the data was used in the reported study.

If your submission does not contain these data, please either upload them as Supporting Information files or deposit them to a stable, public repository and provide us with the relevant URLs, DOIs, or accession numbers. For a list of recommended repositories, please see https://journals.plos.org/plosone/s/recommended-repositories.

If there are ethical or legal restrictions on sharing a de-identified data set, please explain them in detail (e.g., data contain potentially sensitive information, data are owned by a third-party organization, etc.) and who has imposed them (e.g., an ethics committee). Please also provide contact information for a data access committee, ethics committee, or other institutional body to which data requests may be sent. If data are owned by a third party, please indicate how others may request data access.

4. When completing the data availability statement of the submission form, you indicated that you will make your data available on acceptance. We strongly recommend all authors decide on a data sharing plan before acceptance, as the process can be lengthy and hold up publication timelines. Please note that, though access restrictions are acceptable now, your entire data will need to be made freely accessible if your manuscript is accepted for publication. This policy applies to all data except where public deposition would breach compliance with the protocol approved by your research ethics board. If you are unable to adhere to our open data policy, please kindly revise your statement to explain your reasoning and we will seek the editor's input on an exemption. Please be assured that, once you have provided your new statement, the assessment of your exemption will not hold up the peer review process.

**Author Response**: We are happy to comply with the open data policy of PLOS ONE. Could you kindly suggest the statement that best clarifies this?

5. We note that Figures 1 to 4 in your submission contain map images which may be copyrighted. All PLOS content is published under the Creative Commons Attribution License (CC BY 4.0), which means that the manuscript, images, and Supporting Information files will be freely available online, and any third party is permitted to access, download, copy, distribute, and use these materials in any way, even commercially, with proper attribution. For these reasons, we cannot publish previously copyrighted maps or satellite images created using proprietary data, such as Google software (Google Maps, Street View, and Earth). For more information, see our copyright guidelines: <http://journals.plos.org/plosone/s/licenses-and-copyright>.

**Author Response**: The maps in Figure 1, 2 and 4 are constructed from a freely available world shape file (<https://datacatalog.worldbank.org/search/dataset/0038272/World-Bank-Official-Boundaries>). The Maps in Figure 3 is constructed from freely available Landsat satellite imagery from the USA Geological Survey (<https://earthexplorer.usgs.gov/>). These are now indicated in the figure captions.
 
We require you to either present written permission from the copyright holder to publish these figures specifically under the CC BY 4.0 license, or remove the figures from your submission:
 
a. You may seek permission from the original copyright holder of Figures 1 to 4 to publish the content specifically under the CC BY 4.0 license.  
 
We recommend that you contact the original copyright holder with the Content Permission Form (http://journals.plos.org/plosone/s/file?id=7c09/content-permission-form.pdf) and the following text:
“I request permission for the open-access journal PLOS ONE to publish XXX under the Creative Commons Attribution License (CCAL) CC BY 4.0 (http://creativecommons.org/licenses/by/4.0/). Please be aware that this license allows unrestricted use and distribution, even commercially, by third parties. Please reply and provide explicit written permission to publish XXX under a CC BY license and complete the attached form.”
 
Please upload the completed Content Permission Form or other proof of granted permissions as an "Other" file with your submission.
 
In the figure caption of the copyrighted figure, please include the following text: “Reprinted from [ref] under a CC BY license, with permission from [name of publisher], original copyright [original copyright year].”
 
b. If you are unable to obtain permission from the original copyright holder to publish these figures under the CC BY 4.0 license or if the copyright holder’s requirements are incompatible with the CC BY 4.0 license, please either i) remove the figure or ii) supply a replacement figure that complies with the CC BY 4.0 license. Please check copyright information on all replacement figures and update the figure caption with source information. If applicable, please specify in the figure caption text when a figure is similar but not identical to the original image and is therefore for illustrative purposes only.
The following resources for replacing copyrighted map figures may be helpful:
 
USGS National Map Viewer (public domain): http://viewer.nationalmap.gov/viewer/
The Gateway to Astronaut Photography of Earth (public domain): http://eol.jsc.nasa.gov/sseop/clickmap/
Maps at the CIA (public domain): https://www.cia.gov/library/publications/the-world-factbook/index.html and https://www.cia.gov/library/publications/cia-maps-publications/index.html
NASA Earth Observatory (public domain): http://earthobservatory.nasa.gov/
Landsat: http://landsat.visibleearth.nasa.gov/
USGS EROS (Earth Resources Observatory and Science (EROS) Center) (public domain): http://eros.usgs.gov/#
Natural Earth (public domain): http://www.naturalearthdata.com/

[Note: HTML markup is below. Please do not edit.]

Reviewers' comments:

**Reviewer #1**

The authors performed "Trace metals and their human health risks in sesame seeds from the main cultivation areas of Ethiopia" in Journal of PLOS ONE. The methodology is good. I suggest major revisions for the article before its publication the Journal of PLOS ONE. In my opinion, the English language expressions in the manuscript require copyediting. The writing of this manuscript is not easy for readers to understand. Some sentences fail to express the original meaning. I suggest using a professional copyeditor or a native English speaker to copy edit this manuscript.

**Author Response**: We thank Reviewer #1 for the positive review of our manuscript. In response, we have thoroughly revised the manuscript to address language issues.

Abstract:
Abstract section should be clear and concise.
Where is LOD, LOQ, recovery?

**Author Response**: We have revised the abstract and included LOD, LOQ and recovery values.

Key word: Sort by alphabetical order.

**Author Response**: We have sorted the keywords by alphabetical order.

Introduction
The logic of the current introduction should be revised, and I suggest organizing the Introduction section as following order: importance and meanings, previous studies (literature review), the gaps of previous studies, and objectives of this study.

**Author Response**: We have revised the introduction based on the suggestion.

The text has many errors that must be carefully corrected by the author

**Author Response**: We have corrected the introduction by addressing any typographical and language errors.

The novelty of the research is not justified.

**Author Response**: The novelty of this research lies in providing insights into the trace metal content of white sesame seeds from all major sesame-producing regions in Ethiopia, addressing a significant gap in the literature. Unlike previous studies that focused on limited regions, this study examined geographical variations in trace metal levels and evaluated the associated health risks, which are crucial for food safety. This is clearly indicated in the introduction.

Introduction is not including up to date and relevant articles
Please see the following articles and to make the article more complete, they must be added to the text
Assessment of heavy metal content in refined and unrefined salts obtained from Urmia, Iran.
Determination of Element Concentration of Brewed Tea Consumed in Iran Using ICP-OES: A Risk Assessment Study
Assessment of rice marketed in Iran with emphasis on toxic and essential elements; effect of different cooking methods
The concentration and probabilistic health risk assessment of trace metals in three type of sesame seeds using ICP-OES in Iran

**Author Response**: The article titled “The concentration and probabilistic health risk assessment of trace metals in three type of sesame seeds using ICP-OES in Iran” has already been cited in the manuscript, now as reference [21]. We have also added the article “Assessment of rice marketed in Iran with emphasis on toxic and essential elements; effect of different cooking methods” as reference [10].
Methodology
Material and method section is not providing sufficient explanation

**Author Response**: We have revised the Material and Method section.

Complete and accurate data analysis should be mentioned

**Author Response**: We have revised the data analysis section for clarity.

Statistical analysis should be clear and concise.

**Author Response**: We have revised the data analysis section for clarity.

Result and Discussion
Please mention the validation method in the text of the article

**Author Response**: The method validation had been provided in the “Material and method” section, under the subsection “Method validation”.

For the results to be written more carefully, please pay attention to the mentioned content and articles
It is necessary to compare with more studies and summarize the results of other studies.
Recommendations and Future Implications should be clear and concise

**Author Response**: We have compared our results with those reported in the literature to contextualize our findings.

Conclusion
Conclusions should be clear and concise

**Author Response**: We have revised the conclusion for clarity.

Please write the limitations of the study

**Author Response**: We have provide the limitations of the study and a recommendation.

Throughout the text, some words must match the format of the journal.
In general, the text has many errors that must be carefully corrected by the author.

**Author Response**: We have revised the text and corrected errors.

Use newer references

**Author Response**: We have provided newer and relevant references.

**Reviewer #2**

In this article, the concentration of some heavy metals in sesame seeds harvested from different farms in Ethiopia has been investigated. Although the topic of the article is a priority in terms of the importance of food health, but the article has flaws that are not acceptable in my opinion. These are listed below.
1- The introduction does not sufficiently specify the importance and innovation of this article.

**Author Response**: We have revised the introduction to better highlight the importance and novelty of the article.

2- Chemical fertilizers used in different regions are not mentioned in the materials and method section.

**Author Response**: We did not conduct any survey or study to investigate the nature and types of fertilizers used in the studied areas, as this was outside the scope of the research. Our focus was solely on examining the presence or absence of differences in trace metal concentrations in the sesame seeds, which is now clearly stated in the conclusion section.

3- It seems that checking and analyzing the concentration of heavy elements alone in sesame seeds is not enough. It is suggested that the concentration of heavy metals in the soil of the region was also checked.

**Author Response**: We did not investigate the relationship between the concentrations of metals in the sesame seeds and those in the soil, as this was outside the scope of the study. We believe that not all studies on plants require accompanying soil data, as it depends on the study's objectives. Our study focused on identifying variations in trace metal concentrations and assessing the associated health risks of sesame seeds from all commercial production areas in Ethiopia.

4- The results part seems to be more like presenting a scientific report rather than a scientific and research article.

**Author Response**: We have presented and discussed our findings in terms of the trace metal composition of sesame seeds, geographical variations across the study areas, comparison with existing literature, and the implications for health risks. We believe that the presentation meets the standards of a scientific paper.
5- What is the purpose of statistical correlation analysis? What is the conclusion of the presentation of correlation results?

**Author Response**: This is now clearly explained.

6- In Table 9, the correlation analysis results of significant levels are not specified.

**Author Response**: We have indicated the significant levels.

7- It is suggested to use more statistical analyzes such as principal component analysis in addition to correlation to determine the concentration of heavy metals.

**Author Response**: Principal component analysis has been included in the analysis of results.

8- The conclusion should be fundamentally rewritten. The results of this paper should be compared and evaluated with other similar researches in Ethiopia or other regions or other products.

**Author Response**: We have rewritten the conclusion. We had already compared and evaluated with other similar researches in Ethiopia or other countries.

9- What is the reason for the presence of heavy metals in sesame seeds? This is important to discuss.

**Author Response**: Our goal was to investigate the variation in trace metal content in sesame seeds from different cultivation regions of Ethiopia and assess the potential health risks associated with these variations. Determining the precise sources and their respective contributions would require a separate study.

**Reviewer #3**

The manuscript deals with "Trace metals and their human health risks in sesame seeds from the main cultivation areas of Ethiopia" the following comments and suggestions are need to address before its consideration.
1. The abstract should succinctly summarize the key findings, including specific health risks associated with trace metals. Currently, it mentions that non-carcinogenic health risks were not observed, but elaborating on the implications of these findings would strengthen the abstract

**Author Response**: We have indicated the health implications the findings in the abstract.

2. The methodology section should provide more detail on the sampling process, including the number of samples collected from each district and the criteria for selection. This will enhance reproducibility and transparency in the research

**Author Response**: We have provided sufficient details on the sampling process.

3. The introduction highlights the geographical influence on trace metal contents, but the discussion could further explore how specific environmental factors in each district may contribute to the observed differences in metal concentrations. This would provide a more comprehensive understanding of the results

**Author Response**: As described in the manuscript, the differences in trace metal content observed among sesame seeds from different cultivation areas could be attributed to factors such as genetic variation of the plant, environmental factors like soil composition, or agronomic practices, including the type and quantity of fertilizers used. However, investigating the specific contributions of these various potential sources of trace metals is beyond the scope of this study.

4. While the statistical methods used (e.g., one-way ANOVA) are mentioned, a more thorough explanation of how these analyses were conducted and interpreted would be beneficial. Including assumptions of the tests and how they were checked could improve the rigor of the analysis

**Author Response**: We have provided the assumptions of the tests and how they were checked in the section “Data Analysis”.

5. More emphasis on the quality control measures taken during the study, such as the use of blank solutions and recovery studies, would add credibility to the results. This could be integrated into the methodology section. https://link.springer.com/article/10.1007/s12011-024-04234-0,
[`](https://www.sciencedirect.com/science/article/pii/S0278691524003727),

**Author Response**: Information on the use blank solutions is provided in the subsection “FAAS Determination of metals”, and recovery studies in the subsection “Method validation”.

6. 1. What specific AAS techniques were utilized?, 2. How do findings compare to previous studies? 3. What quality control measures were implemented? Referred to below papers and cite them
https://www.sciencedirect.com/science/article/pii/S0946672X24001019, https://www.sciencedirect.com/science/article/pii/S0946672X24000749, <https://www.sciencedirect.com/science/article/pii/S0889157523008177>

**Author Response**: The AAS technique utilized in the study is describe under the subsection “FAAS Determination of metals”. Comparison with literature reports is presented in Table 7. The quality control measures, including the analysis of blank samples alongside the test samples and the evaluation of method validation parameters such as linearity, precision, accuracy, limit of detection, and limit of quantification, are outlined in the manuscript. We have now cited the suggested paper <https://www.sciencedirect.com/science/article/pii/S0946672X24001019>

7. The manuscript includes comparisons with previous studies, but it could benefit from a more systematic comparison of trace metal levels across different countries. This would contextualize the findings within a global framework and highlight the significance of the results

**Author Response**: We have compared the results with previous studies from different countries.

8. The health risk assessment section should include a more detailed discussion on the potential long-term effects of trace metal consumption, particularly for vulnerable populations such as children. This would enhance the relevance of the study to public health

**Author Response**: The study found no non-carcinogenic health risks to humans from the trace metals present in the sesame seeds, as clearly stated in the manuscript. However, discussing the potential long-term effects of trace metal consumption, particularly related to carcinogenic health risks, is beyond the scope of this study and therefore is not relevant.

9. Incorporating graphs or tables to visually represent the concentration levels of trace metals across different regions would improve the readability and impact of the findings. Visual aids can help convey complex data more effectively

**Author Response**: The data are presented in tables and discussed in the text, as we believe is most appropriate. Repeating the information in figure form would create redundancy.

10. The conclusion should not only summarize the findings but also suggest practical implications for consumers and policymakers regarding sesame seed consumption and agricultural practices. This would provide actionable insights based on the research

**Author Response**: We have revised the conclusion.

6. PLOS authors have the option to publish the peer review history of their article ([what does this mean?](https://track.editorialmanager.com/CL0/https:%2F%2Fjournals.plos.org%2Fplosone%2Fs%2Feditorial-and-peer-review-process%23loc-peer-review-history/1/010f019307fe1704-00063818-564f-4f39-83c4-5b133d7a9f07-000000/8gelXQe40vVpxFgXhYZ47eXXZ46RfNP3E0Nf7uOOXEg=184)). If published, this will include your full peer review and any attached files.


If you choose “no”, your identity will remain anonymous but your review may still be made public.


**Do you want your identity to be public for this peer review?** For information about this choice, including consent withdrawal, please see our [Privacy Policy](https://track.editorialmanager.com/CL0/https:%2F%2Fwww.plos.org%2Fprivacy-policy/1/010f019307fe1704-00063818-564f-4f39-83c4-5b133d7a9f07-000000/1poWGTRZEt0szH4QUcziYLchoI4Y0h-QBaSY5pr0rBc=184).

Reviewer #1: No

Reviewer #2: No

Reviewer #3: No
